# Multigenerational effects of bisphenol A or ethinyl estradiol exposure on F_2_ California mice (*Peromyscus californicus*) pup vocalizations

**DOI:** 10.1371/journal.pone.0199107

**Published:** 2018-06-18

**Authors:** Sarah A. Johnson, Michelle J. Farrington, Claire R. Murphy, Paul D. Caldo, Leif A. McAllister, Sarabjit Kaur, Catherine Chun, Madison T. Ortega, Brittney L. Marshall, Frauke Hoffmann, Mark R. Ellersieck, A. Katrin Schenk, Cheryl S. Rosenfeld

**Affiliations:** 1 Bond Life Sciences Center, University of Missouri, Columbia, Missouri, United States of America; 2 Department of Biomedical Sciences, University of Missouri, Columbia, Missouri, United States of America; 3 Department of Animal Sciences, University of Missouri, Columbia, Missouri, United States of America; 4 Department of Gastroenterology, School of Medicine, University of Missouri, Columbia, Missouri, United States of America; 5 Department of Chemicals and Product Safety, The German Federal Institute for Risk Assessment, Berlin, Germany; 6 Department of Agriculture Experimental Station-Statistics, University of Missouri, Columbia, Missouri, United States of America; 7 Department of Physics, Randolph College, Lynchburg, Virginia, United States of America; 8 Genetics Area Program, University of Missouri, Columbia, Missouri, United States of America; 9 Thompson Center for Autism and Neurobehavioral Disorders, University of Missouri, Columbia, Missouri, United States of America; McGill University, CANADA

## Abstract

Rodent pups use vocalizations to communicate with one or both parents in biparental species, such as California mice (*Peromyscus californicus*). Previous studies have shown California mice developmentally exposed to endocrine disrupting chemicals, bisphenol A (BPA) or ethinyl estradiol (EE), demonstrate later compromised parental behaviors. Reductions in F_1_ parental behaviors might also be due to decreased emissions of F_2_ pup vocalizations. Thus, vocalizations of F_2_ male and female California mice pups born to F_1_ parents developmentally exposed to BPA, EE, or controls were examined. Postnatal days (PND) 2–4 were considered early postnatal period, PND 7 and 14 were defined as mid-postnatal period, and PND 21 and 28 were classified as late postnatal period. EE pups showed increased latency to emit the first syllable compared to controls. BPA female pups had decreased syllable duration compared to control and EE female pups during the early postnatal period but enhanced responses compared to controls at late postnatal period; whereas, male BPA and EE pups showed greater syllable duration compared to controls during early postnatal period. In mid-postnatal period, F_2_ BPA and EE pups emitted greater number of phrases than F_2_ control pups. Results indicate aspects of vocalizations were disrupted in F_2_ pups born to F_1_ parents developmentally exposed to BPA or EE, but their responses were not always identical, suggesting BPA might not activate estrogen receptors to the same extent as EE. Changes in vocalization patterns by F_2_ pups may be due to multigenerational exposure to BPA or EE and/or reduced parental care received.

## Introduction

Diverse rodent species primarily communicate by ultrasonic vocalizations (USVs, sounds above 20 kHz). We, and others, have characterized USV production by neonatal California mice (*Peromyscus californicus*) and other rodent species [1–7]. Pup vocalizations may be essential in triggering parental care [2, 5, 8]. This may be especially important in those that are unable to regulate their own body temperature until later in life, which includes neotropical singing mice (*Scotinomys spp*.) [4] and California mice [9–11]. The latter species are monogamous and biparental [10, 12–14], and while the dam is foraging, her male partner will huddle over the pups to prevent a decline in pup body temperature [9, 10]. We have previously shown that California mice pup vocalizations begin to decline around the time, postnatal day (PND) 15, they can regulate their own body temperature.

In our past study, we have shown that male and female California mice developmentally exposed to the endocrine disrupting chemicals (EDCs), bisphenol A (BPA), which can exhibit weak estrogenic activity or ethinyl estradiol (EE, estrogen present in birth control pills and estrogen receptor positive control for the current studies) demonstrate reduced biparental care, including duration of time female spent nursing the pups, time spent in the nest for both parents, and time spent grooming the pups [15]. While the reduced parental care by the F_1_ parents developmentally exposed to BPA or EE could be due to neurobehavioral disruptions in these individuals, it could also be attributed to decreased vocalizations emitted by their F_2_ offspring exposed multigenerationally to these chemicals. Developmental exposure of Sprague-Dawley rats to polychlorinated biphenyls (PCBs, Aroclor 1221 PCR mixture) disrupt the number of vocalizations produced by sexually mature female rats [16]. It has also recently been reported that maternal exposure of C57BL/6J mice to 20 μg BPA per day via a chocolate-flavored treat one week prior to breeding and throughout gestation increased the duration and median frequency of USVs emitted by F_1_ pups at PND 8 during maternal separation [17]. Several EDCs, vinclozolin, EE, p,p'-Dichlordiphenyldichloroethylene (p,p'-DDE), Levonorgesterel (synthetic progesterone) disrupt mating calls produced by male South African clawed frogs (*Xenopus laevis*) [18–22]. Zebra finch (*Taeniopygia guttata*) male and female progeny derived from females exposed to PCB, Aroclor 1248, prior to egg-laying have significant reduction in the song control nuclei robustus arcopallialis (RA) or now called the robust nucleus of the arcopallium, which could compromise their song production ability [23]. Similarly, treatment of adult male starlings (*Sturnus vulgaris*) with estrogenic EDCs was shown to result in louder, longer and more complex songs compared to vocalizations of control males [24]. These effects were suggested to be due to an increase in volume of the principle nucleus in the songbird brain, the HVC(used as proper name) [24].

Based on these above studies showing direct exposure to various EDCs in a variety of taxa or exposure of F_1_ rodent pups to the EDCs, BPA or Aroclor 1221 PCR mixture, alters vocalization patterns [16–24], we hypothesized that audible and ultrasonic vocalizations might be reduced in F_2_ pups multigenerationally exposed to BPA or EE, which could partially account for the reduced parental care provided by their F_1_ parents. Another possibility that was explored in the current study is whether pup vocalizations are compromised in F_2_ pups derived from F_1_ offspring developmentally exposed to these EDCs and who demonstrate decreased parental care to their F_2_ pups. In other words, we sought to examine whether F2 pups who are not directly exposed to BPA or EE, demonstrate changes in vocalization parameters. The current studies thus are the first to examine vocalization behaviors in F2 offspring of ancestors directly exposed to BPA or EE. However, the current studies were not aimed at determining the underlying causes (reduced F_1_ parental care and/or multigenerational exposure to BPA or EE) of potential alterations in F_2_ acoustic patterns. To test whether vocalization patterns of F_2_ pups derived from F_1_ pairings of BPA- or EE-exposed parents were disrupted compared to those born to control F_1_ parents, pups were examined from postnatal day (PND) 2 to 28. Additionally, we examined potentiation behavioral responses in the tested pups. In this case, a potentiated behavioral response refers the to the fact that a pup who has already been isolated may vocalize more after being returned to their parents and then isolated again, in essence this behavioral response is even greater after being isolated and tested a second time [7, 25–27]. Mid postnatal age rat pups returned to a passive (anesthetized dam) show increased vocalization responses when isolated again, which is considered a potentiation response in that re-isolation after being placed back with one or both parents causes an enhanced response relative to the vocalization responses when initially isolated from one or both parents [25, 27]. No maternal potentiation effects were observed on PND 8 in mice developmentally exposed to BPA [17]. Passive (anesthetized) and active maternal potentiation but not paternal potentiation has been reported in prairie voles (*Microtus ochrogaster*), which are also monogamous and biparental [28]. In the current studies, California mice pups from all treatment groups were returned to their dam and sire, and thus, the current studies tested whether a combination of maternal and paternal potentiation exists and whether it can be affected by multigenerational exposure to BPA or EE in F_2_ pups.

## Materials and methods

### Animal husbandry and treatments

Founder outbred adult (60–90 days of age) male and female California mice that were pathogen-free, were purchased from the *Peromyscus* Genetic Stock Center (PGSC) at the University of South Carolina (Columbia, SC). Upon arrival, individuals were placed in quarantine at the University of Missouri for a minimum of 8 weeks to verify their disease-free status. All experiments were approved by University of Missouri Animal Care and Use Committee (Protocol #8643) and performed in accordance with the recommendations in the Guide for the Care and Use of Laboratory Animals of the National Institutes of Health. Two weeks prior to breeding, virgin P_0_ females, 8 to 12 wks of age were randomly assigned to receive one of three diets: 1) a low phytoestrogen AIN 93G diet supplemented with 7% by weight corn oil to minimize potential phytoestrogenic contamination that would otherwise be present with inclusion of soybean oil in the diet (control), 2) this diet supplemented with 50 mg BPA/kg feed weight, which we have documented to lead to internal serum concentrations close to those measured in pregnant women unknowingly exposed to this chemical [29, 30], and 3) AIN93G diet supplemented with 0.1 parts per billion of EE, as the FDA required positive control for BPA studies based on the fact that BPA is considered a weak estrogen [31]. Additionally, EE-exposed individuals might provide insights into whether BPA effects might be due to BPA binding and activating estrogen receptors (ESRs) to the same extent as EE. We have previously validated similar effects of the chosen BPA and EE doses in deer mice (*P*. *maniculatus bairdii*) [29, 32] and California mice [15, 33–35]. The P_0_ dams remained on the diet throughout gestation and lactation, as described previously [29, 32–36]. The F_1_ generation sons and daughters were weaned at 30 days of age, singly housed and placed on the AIN93G (control) diet. Food and water were provided *ad libitum*.

When F_1_ animals reached adulthood (~90 days of age), males and females from each group were paired with respective partners who were developmentally exposed to the same diet, such that there were three F_1_ breeding pairs: BPA-exposed for males and females, EE-exposed for males and female, and AIN93G control for males and females. When individuals were not in the Phenotyper system (Noldus Technologies, Leesburg, VA), they were housed in white polypropylene cages (27.8 x 7.5 x 13 cm) and maintained on a 12:12 h light:dark cycle (lights on at 7:00 A.M. CST, lights off at 7:00 P.M. CST). As *Peromyscus* species do not form a visible plug, F_1_ females were weighed weekly to provide some idea of when they might give birth. The day of birth was considered PND 0.

### F_2_ pup ultrasonic vocalization measurements and potentiation experiments

Each individual pup was tested in isolation and away from their home-cage, as we have described previously [7]. The total number of individual pups and litters tested are indicated in Table 1. This number of pups and litters is similar to previous studies that have examined the effects of developmental exposure of other rodents to the EDCs, Aroclor 1221 PCR mixture and BPA [16, 17]. Pup USVs and potentiation experiments were initiated on PND 2. Each pup was demarcated with a distinguishing tattoo mark on either the front or back paw (Fine Science Tools, Foster City, CA). California female mice can only nurse up to four pups with an average litter size of 2.2, and pups are weaned at 30 days of age [7, 10, 15]. All F_2_ pups in the litter were recorded throughout the entire postnatal period (PND 28), assuming they survived up until this time-point. Pup recordings were performed at ~10.00 hrs (3 hrs after lights turned on in the room) and 14.00 hrs (7 hrs after lights turned on in the room). Before testing, the experimenter noted whether they were or were not latched on to one of the dam’s four teats (S1 Table). The pups were placed individually on a laminated sheet in a polypropylene box (22 inches by 27 inches) surrounded on three sides with a 20 inch by 20 inch black polypropylene box lined with two inches of convoluted acoustic foam panel (SoundproofCow, Chambersburg, PA), as depicted in our previous study [7]. The sheet demarcated pup and litter information, date, and time of day when testing was done and was disinfected with 70% ethanol between trials. No substrate material, including shavings or bedding material, was present in the recording box. The box was equipped with an Avisoft Bioacoustics CM16/CMPA40-5V microphone (Glienicke, Germany) plugged into an National Instruments USB 6351 data collection board hooked up to a Dell computer (Roundrock, TX). USV data were collected using a custom LabView data collection code written by the laboratory of Dr. Katrin Schenk at Randolph College. A Logitech Carl Zeiss Tessar HD 1080P (Newark, CA) camera was mounted onto a Joby- Gorilla Pod Original Tripod (Daymen US Inc., Petaluma, CA) in the recording box to monitor isolated pups. Pups were audio-recorded for three minutes, which minimized the time away from their parents but permitted relevant data to be collected for the time period assessed. The pup was then placed back in the home cage with both parents for five minutes. All pups within a litter were recorded for a given day before testing the next litter. However, the order of testing was randomized between treatments such that pups within the same litter were not always tested first or last on a given day.

**Table 1:** Total number of F_2_ male and female pups and number of litters examined for neonatal pup vocalization studies.

Treatment Group	# of Male Pups Tested	# of Female Pups Tested	# of Pups with Undetermined Sex*	Total # of Pups Examined	Total # of Litters
**Control**	3	3	4	10	8
**BPA**	6	10	2	18	12
**EE**	9	7	7	23	13

*At the outset of the current studies, pup sex was not initially considered.

After being returned to the home cage, the pup was isolated again and returned to the audio-recording box and its vocalizations recorded for another three minutes as part of a combination maternal and paternal potentiation experiment. The audio-recording box was sprayed down between trials with 70% ethanol for disinfection and to remove any potential confounding olfactory cues for subsequent pups tested. Pup vocalizations were measured on PND (age of the pups) 2, 3, 4, 7, 14, 21, and 28. The previous study examining effects of developmental exposure to BPA on mouse pups only recorded them at PND 8 [17]. However, as California mice pups are hypothermic up until about PND 15 [9–11], we sought to examine the F_2_ vocalization responses from the early PND to the late PND to determine how exposure to BPA or EE might affect the full gamut of their vocalization responses from this period, as we had previously done when characterizing the ontogenic profile of vocalization responses in control California mice pups [7] and has been done with mandarin vole (*Microtus mandarinus*) pups, where the parents are also monogamous and biparental [37]. Recordings on PND 2–4 were considered early postnatal period, those on days 7 and 14 were deemed mid-postnatal period, and those on days 21 and 28 were considered late postnatal period. These time-points were chosen to span the early, mid, and late postnatal periods and key milestones in California mice neonatal development. Based on our observations, the fur begins to emerge and darken between two to four days of age. Eyes open around 14–15 days of age in this species, which is also when they can begin to thermoregulate on their own [7, 9, 10, 38, 39]. California mice pups begin walking and rearing around 22 and 22.75 days of age [38]. Pups raised by both parents begin self-grooming around 25 days of age. California mice pups are weaned at 30 days of age and eat solid food at this time [10].

Vocalizations throughout the entire duration of recording (3 minutes for both trials) were detected and measured by a MATLAB-based program developed by our collaborator and coauthor (Dr. A. Katrin Schenk), which allows USVs up to 200 KHz to be detected and analyzed, as described previously [7]. Briefly, for each recording, all syllables were segmented for analysis. The experimenter was blind to treatment when denoising and segmenting the calls. Syllables are synonymous with calls, as defined by other rodent EDC studies [16, 17]. Number of syllables, syllable duration, syllable’s mean median frequency, average syllable power (a summation of power percent below and above 20 kHz), power percent below 20 kHz, and power percent above 20 kHz were determined. The average power was calculated as follows: 1) For each time bin in the short-time Fourier transform (spectrogram) of the syllable determined the mean power in units of decibels above the noise level. 2) The mean of these values for all time bins contained in the syllable’s spectrogram was taken. The noise level used in step one was calculated in the following way: 1) A 2 second region of the raw data spectrogram was picked as a representative of the background noise. This region was identified by examining the spectrogram by eye to make sure that there were no syllables or artifacts present. 2) The spectrogram of this region was averaged over all time bins to create a baseline noise per frequency bin. 3) Then the noise level per frequency was defined as 3 standard deviations about the baseline noise. Thus, the average power is a measure of intensity. The median frequency per call was averaged for each pup, hence the mean of the median frequencies. The median frequency was chosen per Liu et al 2003 [40] as a read out of the spectral content of the call. While there are many different read outs that one might choose, we elected to use the median frequency since it takes into account all components of the call (S1 Fig).

**Fig 1:**
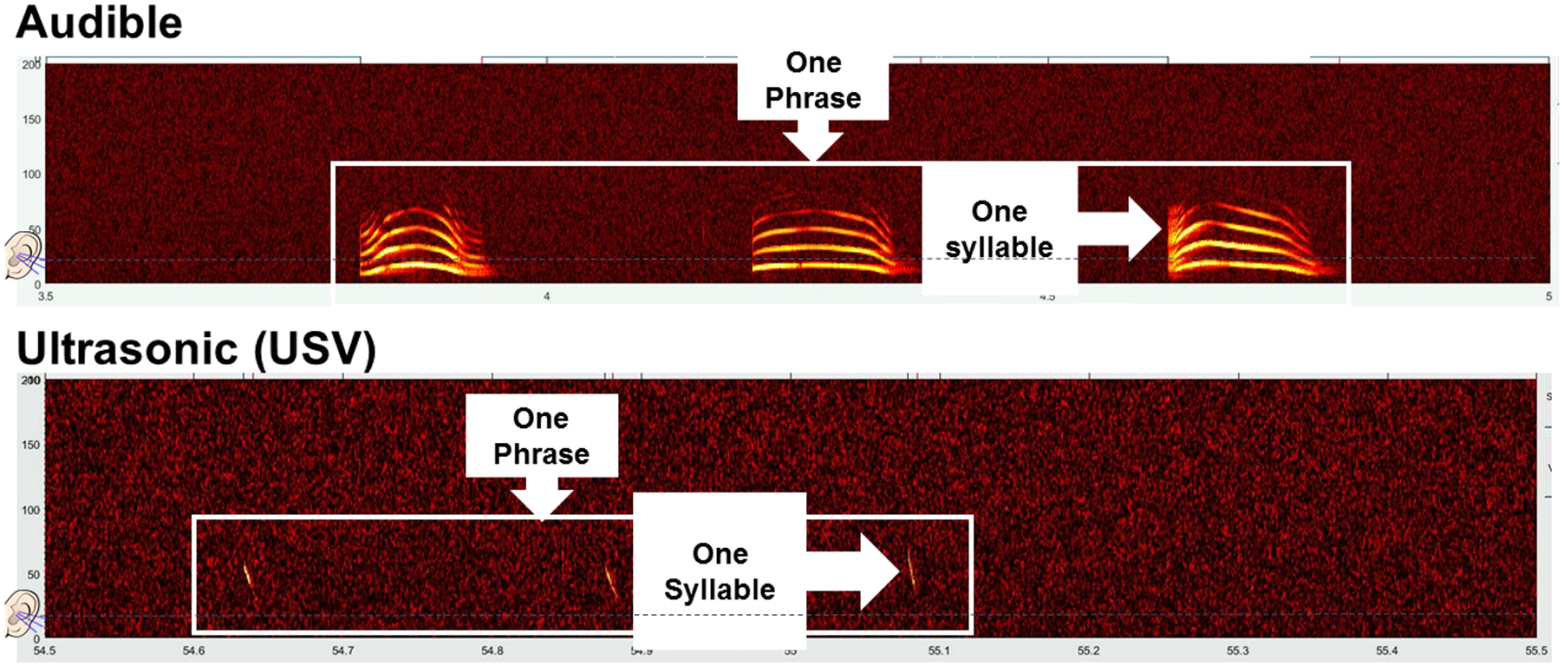
Example audible and ultrasonic vocalizations in California mice pups. The top panel depicts syllables, which are the same thing as calls, that begin in the audible range of below 20 kHz and then extend into the ultrasonic range. The bottom panel depicts USVs alone that are above 20 kHz. A phrase or burst is a group of syllables separated from another group of syllables by an interval in time that is statistically longer than the mean time between individual syllables. The ear lobe at the left side of each panel represents the fact that human hearing only extends up to 20 kHz.

Additionally, the number of phrases, which designates a group of syllables separated from another group of syllables by an interval in time that is statistically longer than the mean time between individual syllables, duration of phrases, and number of syllables in a phrase were measured. Phrase is equivalent to what has also been called a burst in a study examining the effects of developmental exposure to BPA on mouse vocalizations [17]. The terminology of syllables and phrases is used herein to be consistent with previous studies that have measured vocalizations in California mice [3, 6, 7, 41]. Example syllables and phrases in California mice are shown in Fig 1. Additional representative audible and ultrasonic vocalizations in neonatal California mice pups are shown in [7].

### Statistics

Data were analyzed by using SAS version 9.4 Software (SAS Institute, Cary, NC). Partial eta squared values (Ƞ^2^_p_) were calculated as measure of effect size. A split plot in space and time (an ANOVA-based method) was used to analyze the data, as detailed by [42]. The main plot consisted of F_1_ maternal/paternal treatment, and the subplot was F_2_ pup sex and F_1_ parental treatment X F_2_ pup sex. S2 Fig provides a diagram of how the split plot in space and time controls for litter effects (split plot in space) and repeated measure analyses (time). For pups where the sex was not determined, they were only considered in main plot. Additionally, the sub-sub plots consisted of postnatal period (early, mid, or late), time of day (AM or PM), and trial (Trial 1 or Trial 2) and all possible interactions with these variables and the main plot and sub-plot effects. We sought to analyze the F_2_ pup vocalization responses over the entire course of the post-natal period and in the AM and PM to determine whether the maternal/paternal treatments altered the pattern of the vocalization responses as the pups matured and to examine for potential circadian differences, as it relates to maternal/paternal treatment. In a previous study with F_1_ California mice, we found that developmental exposure to BPA altered later voluntary physical activity predominantly during the dark but not the light cycle [35]. Thus, we postulated that we might detect similar circadian differences in terms of F_2_ pup vocalization responses. Mean differences were determined using Fisher’s Protected Least Significant Difference (LSD), as described by [42]. In this case, the LSD is only calculated if the overall F test is significant and is considered an acceptable method under these conditions [43, 44]. Moreover, we only ran pre-planned and relevant comparisons where pup sex, day, time, and/or trial were hold constant, and the only thing varied was F_1_ maternal/paternal treatment. Additionally, only the highest order of interactions with maternal/paternal treatment was considered. Both of these approaches reduced the number of comparisons and likelihood of committing a Type I error. We decided to use the Fisher’s protected LSD, as recommended by [43, 44], as other methods, including Bonferroni, may mask real differences, and thereby, increase the likelihood of committing a Type II error. The Fisher’s protected LSD helps avoid committing a type I error but at the same time reducing the potential of committing a type II error. With the split plot design, change in the standard error of the differences reflects more than one error term (as detailed by [45]), which is taken into account with Fisher’s protected LSD. Additionally, to test for normality, we analyzed ranked data, which are the only ones presented in the Results section. All data are presented though as actual means ($\overset{-}{x}$) rather averaged rank data. The error bars for all figures and reported data represent the standard error of the mean (SEM).

## Results

### Syllable results

**Fig 2:**
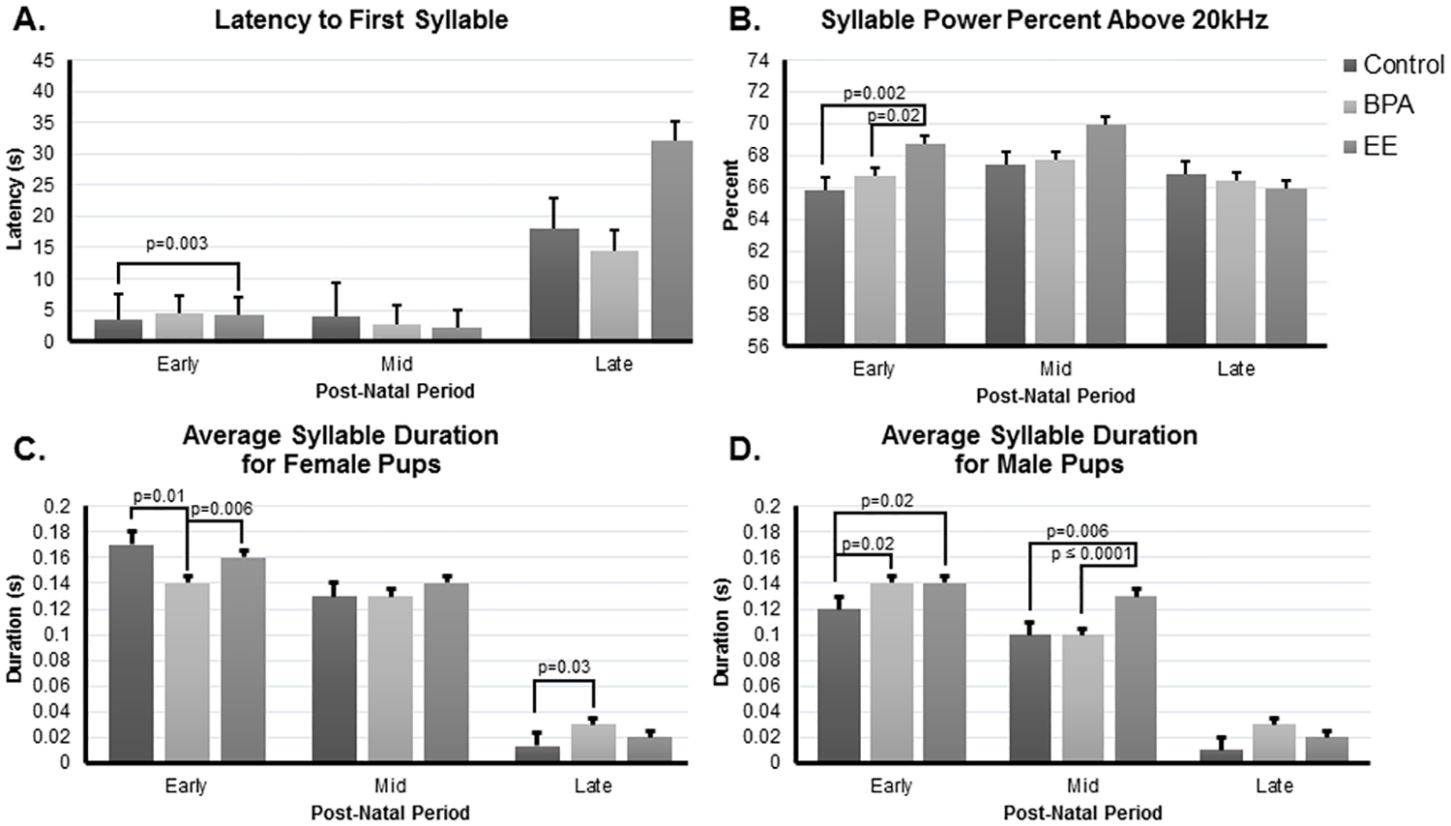
Syllable parameter results in F_2_ pups. A) Latency to first syllable. B) Syllable Power Percent Above 20 KHz. C) Average syllable for female pups. D) Average syllable duration for male pups. Error bars represent SEM.

The first variable considered was latency to produce the first syllable (otherwise considered a call in [17]). A two-way interaction existed between maternal/paternal treatment (main effect) and day (Fig 2A, η^2^_p_ = 0.95, p = 0.0002). Latency to first syllable in the early postnatal days was greater in EE compared to control pups (p = 0.003). Likewise, the percentage of syllable power below and above 20 kHz showed a two-way interaction with maternal/paternal treatment (main effect) and day (Fig 2B, η^2^_p_ = 0.94, p ≤ 0.0001). During the early postnatal period, syllable power above 20 kHz was greater in EE compared to control and BPA pups (p = 0.002 and 0.02, respectively, Fig 2B). During those postnatal days, the opposite was the case for syllable power below 20 kHz with control and BPA pups showing greater syllable power than EE individuals (p = 0.002 and 0.02, respectively).

Syllable duration demonstrated a three-way interaction with maternal/paternal treatment (main effect), day, and pup sex (Fig 2C & 2D, η^2^_p_ = 0.98, p ≤ 0.0001). During the early postnatal period, female control and EE pups had longer syllable duration than female BPA pups (p = 0.01 and p = 0.006, respectively Fig 2C). However, by the late postnatal period, BPA females exhibited longer syllable duration than control females (p = 0.03, respectively). In the early period, BPA and EE males had greater syllable duration than control males (p = 0.02 for both, Fig 2D). In the mid-period, EE males continued to exhibit increased syllable duration relative to control and BPA males (p = 0.006 and ≤ 0.0001, respectively, Fig 2D).

**Fig 3:**
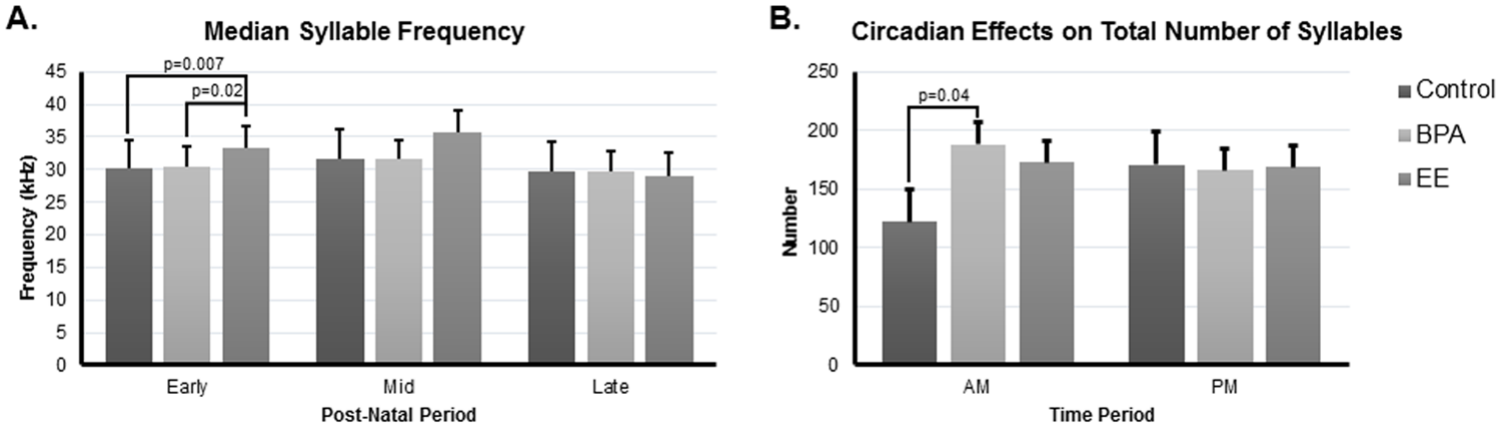
Other syllable parameter results in F_2_ pups. A) Median syllable frequency. B) Circadian effects on total number of syllables. Error bars represent SEM.

The mean of the median frequencies of the syllables was significantly affected by the interaction of maternal/paternal treatment (main effect) and day (Fig 3A, η^2^_p_ = 0.96, p ≤ 0.0001). In the early postnatal period the median syllable frequency was greater in EE compared to control and BPA pups (p = 0.007 and 0.02, respectively, Fig 3A). Total number of syllables was altered by maternal/paternal treatment (main effect) and time of day (Fig 3B, η^2^_p_ = 0.96, p = 0.03). In the morning, BPA pups called more than controls (p = 0.04, Fig 3B). None of the other syllable variables showed significant maternal/paternal treatment differences.

### Phrase results

**Fig 4:**
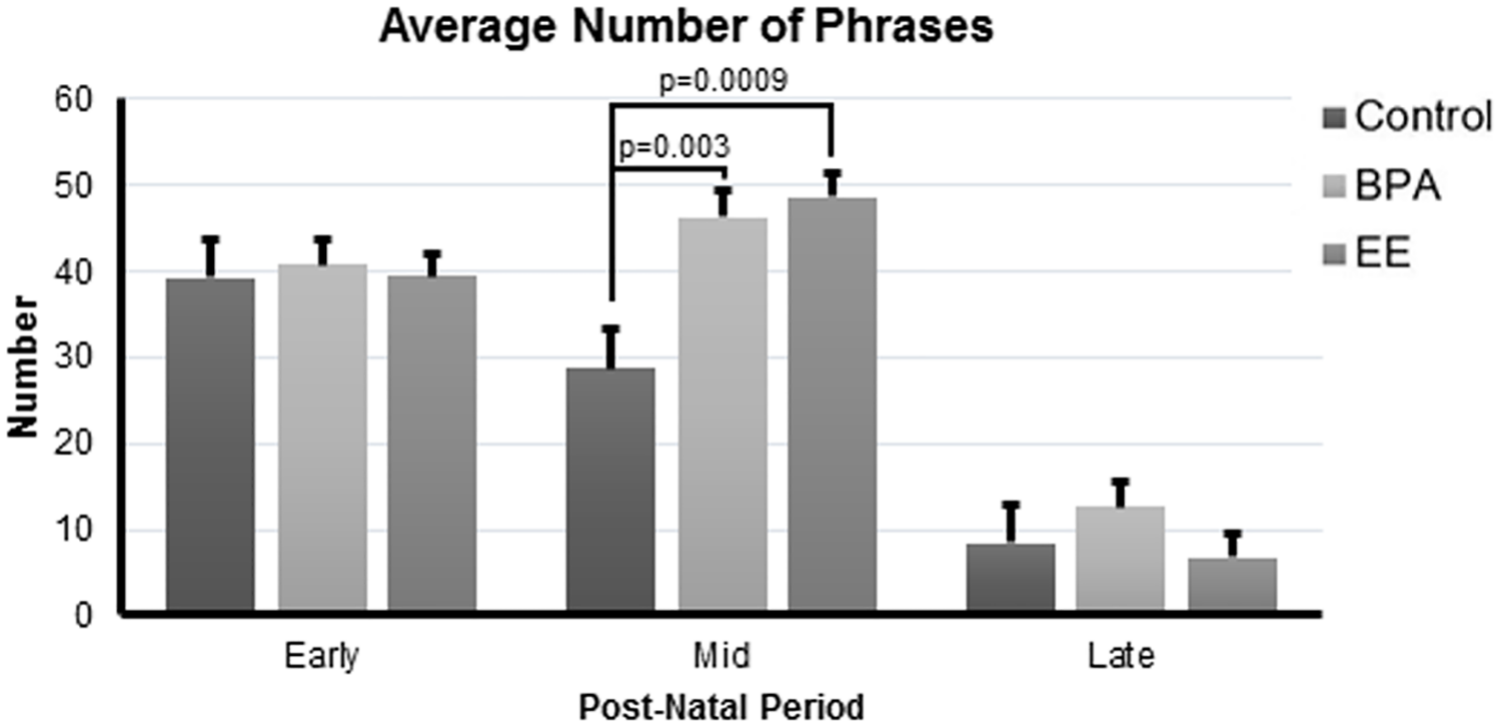
Average number of phrases in F_2_ pups. Error bars represent SEM.

The average number of phrases (also called a burst in [17]) was influenced by the interactions of: 1) maternal/paternal treatment (main effect) and day and 2) maternal/paternal treatment (main effect) and day (η^2^_p_ = 0.96, p = 0.002 and η^2^_p_ = 0.96, p = 0.04, respectively). In the mid-postnatal period, BPA and EE pups had greater average number of phrases than control individuals (p = 0.003 and 0.0009, respectively, Fig 4). Circadian differences were also noted between these groups with this category of vocalizations being greater in the morning for BPA and EE pups compared to control pups (p = 0.01 and 0.03, respectively, S3 Fig). None of the other phrase variables showed significant maternal/paternal treatment differences.

### Examination of possible maternal and paternal potentiation effects

Potentiation experiments were also performed to test whether the pup responses were greater after being isolated and tested in Trial 1, then placed back with both parents, and then re-isolated again to measure their vocalization responses for Trial 2. No differences in any of the vocalizations parameters measured though showed potentiation differences, which is similar to data observed when comparing F_1_ BPA-exposed and control mice [17].

## Discussion

The current studies tested the hypothesis that F_2_ California mice pups born to F_1_ parents developmentally exposed to BPA or EE might emit reduced vocalizations. Potential reductions in F_2_ pup vocalizations might partially account for reduced biparental care previously identified in F_1_ California mice developmentally exposed to BPA or EE [15]. However, vocalization parameters that differed in BPA and EE pups were generally enhanced relative to control counterparts, but temporal and sex differences were noted. While BPA female pups had increased syllable duration compared to control pups during the late postnatal period, they showed reduced response during the early postnatal period. In the mid-postnatal period, BPA and EE pups emitted greater number of phrases than respective control pups. F_2_ BPA and EE pups emitted greater number of phrases in the morning compared to F_2_ control pups. These changes suggest that the reduced parental care provided early on by F_1_ BPA and EE parents resulted in them emitting greater vocalizations, including syllables and phrases at later postnatal periods.

The primary vocalization differences detected in the current studies between F_2_ BPA or EE pups compared to control pups are summarized in Table 2. These F_2_ communication responses might be due to direct effects of multigenerational exposure to these EDCs as the primordial germ cells that gave rise to the F_2_ generation would have been simultaneously exposed when the P_0_ parents consumed the diets and the F_1_ generation was developing (as shown in Figure 11.1 in [46] and other relevant reviews [47–50]). Other studies have shown that multigenerational exposure to BPA can lead to neurobehavioral, reproductive, and cardio-metabolic changes in F_2_ rodent offspring [51–58].

**Table 2:** Summary of vocalization parameters affected in F_2_ pups multigenerationally exposed to BPA or EE compared to control pups.

Vocalization Parameter	Key Findings
**Latency to emit first syllable**	Early postnatal period: EE pups> control pups
**Syllable duration**	Early postnatal period: control and EE females > BPA females. Late postnatal period: BPA females > control females Early postnatal period: BPA and EE males >control males. Mid postnatal period: EE males > control and BPA males
**Median Call Frequency**	Early postnatal period: EE pups > control and BPA pups
**Total number of syllables**	AM: BPA pups > controls pups
**Percentage of syllable power below and above 20 kHz**	Early postnatal period: EE pups > control and BPA pups for those above 20 kHz Early postnatal period: control and BPA pups > EE pups for those below 20 kHz
**Average number of phrases**	Mid postnatal period: BPA and EE pups > control pups AM: BPA and EE pups >control pups

Another possible but not mutually exclusive explanation is that in response to the reduced parental care provided by their F_1_ parents, the F_2_ pups had augmented vocalization responses in attempt to attract their parents to increase their parental investment. The enhanced vocalization responses even up into the late postnatal period, when the pups can thermoregulate and begin to feed on their own might also suggest increased anxiogenic behaviors. Pup body weight might also affect acoustic parameters with heavier pups presumably being able to generate increased magnitude of vocalization responses. While we did not measure F_2_ pup weight in the current studies, we had done so in previous studies [15]. We found that F_2_ pups of F_1_ parents developmentally exposed to BPA had no difference in body weight gain compared to F_2_ pups of F_1_ control parents. However, F_2_ pups of F_1_ parents developmentally exposed to EE showed reduce weight gain from PND 13–32. Yet, in the current studies F_2_ EE females and males showed increased magnitude of vocalization responses, suggesting body weight was not a contributory factor to treatment differences observed in this group. In mandarin voles, which are also monogamous and biparental, there is surprisingly a positive relationship between pup USV emissions and level of parental care received by pups [37]. Follow up studies are thus needed to determine whether various behavioral responses, including vocalization, anxiogenic, cognitive, and sociosexual behaviors continue to be affected as the F_2_ pups mature.

To tease apart whether the postnatal vocalization responses in the F_2_ pups are multigenerational in origin or due to poor parental care provided by F_1_ parents, cross-fostering approaches might be useful. This procedure was not done in these initial studies, as we were concerned whether the foster parents would successfully rear the pups. Additionally, we wished to use the same approach where reduced parental care was identified in F_1_ BPA and EE exposed parents [15]. Such future cross-fostering studies would entail placing control F_2_ pups with F_1_ BPA or EE exposed parents and F_2_ BPA/EE pups with F_1_ control parents. If the elevated vocalization responses in F_2_ BPA and EE pups were multigenerational in origin, the prediction would be that they would continue when placed with control parents. However, if they originate due to receiving inadequate parental care by their F_1_ exposed parents, then F_2_ BPA and EE pups should have reduced vocalizations when placed with control F_1_ parents who will presumably be more attentive.

The expectation at the outset was that similar vocalization responses would be observed in F_2_ BPA or EE pups. While such was the case for select parameters measured, F_2_ EE pups differed from both control and BPA counterparts for various vocalization aspects, such as latency to emit first syllable, median call frequency, percentage of syllable power below and above 20KHz. The more pronounced effects in the EE group might be attributed to differences in estrogen receptor binding and activation, as BPA is considered a weak-estrogen [59, 60]. It might also suggest that multigenerational exposure to BPA and EE can result in differing effects. In our study examining effects of developmental exposure to BPA or EE on biparental behaviors in California mice, both chemicals led to similar disruptions for nursing behaviors in the case of the dams and time spent in the nest for both parents [15]. However, those exposed to EE groomed their F_2_ pups more relative to control parents with control F_2_ pups (as determined based on measuring the frequency and duration of grooming from archived videos taken with an infra-red camera/ Phenotyper (Noldus Technologies, Leesburg, VA), that recorded at several time-points throughout the light and dark cycle and on several days spanning from PND 2 to PND 30), but such responses were not observed in those exposed to BPA. In other studies examining neurobehavioral disruptions induced by developmental exposure to BPA and EE in California mice, deer mice (*P*. *maniculatus bairdii*), Sprague-Dawley rats, and painted turtles (*Chrysemys picta*), we have found that these EDCs do not always induce similar gene expression changes in the brain or behavioral alterations [29, 32–36, 61, 62]. Thus, the current data suggest that EE may not be the ideal positive control for BPA developmental exposure studies and other steroidogenic compounds, such as androgens, should possibly be tested as well.

Another prediction at the outset was that F_2_ BPA and EE exposed male and female pups would should similar vocalization changes. However, sexually dimorphic differences were observed for syllable duration. While BPA females showed reductions in this category during the early postnatal period relative to control and EE females, BPA and EE males showed enhanced response relative to controls. BPA females demonstrated increased syllable duration compared to controls during the late postnatal period, but no differences were detected at this time period between BPA and control pups. Other studies indicate that wild-type and transgenic rodents demonstrate sex differences in vocalization patterns at various postnatal ages. For instance, male rat pups placed in isolation elicit substantially more USV calls that were typified by a significantly lower frequency and amplitude compared to isolate female rat pups [63]. This same study also showed that dams are more likely to retrieve and place male pups back into the nest than females. Testing of vocalization patterns of fragile X mental retardation 1 (*Fmr1*) knockout male and female mice from postnatal days 9–14 reveals that males and females diverge in the total number of calls and call duration depending on the postnatal day [64], which is similar to the results identified in the current study with F_2_ BPA female and male pups.

To date, two other studies have examined the effects of exposure to EDCs on rodent vocalizations. Developmental exposure to the PCB mixture, Aroclor 122, disrupts the number of vocalizations produced later on by sexually mature adult female Sprague-Dawley rats [16]. Maternal exposure of C57BL/6J mice to 20 μg BPA per day one week prior to breeding and throughout gestation increased the duration and median frequency of USVs, as well as the total number of “bursts” (which is the same as phrases) emitted by F_1_ pups during maternal separation when tested at PND 8 [17]. However, in this study, the number of calls was not altered by *in utero* exposure to BPA. The initial study testing the effects of PCBs on rat vocalizations was done prior to some of the recent advancements in detecting and analyzing rodent vocalization, and thus, EDC-induced disturbances on other facets of both audible and ultrasonic vocalizations might have been missed. The latter study with F_1_ mice pups used an identical microphone and software program as that used herein. Notably, BPA exposure of F_1_ pups in the Harris et al study [17] could have induced direct effects on brain development in these pups that might have in turn affected their vocalization responses. However, the developing brain and other organs of F_2_ pups were not directly exposed to BPA but only multigenerationally exposed to BPA via the primordial germ cells contained within the developing F_1_ offspring (as shown in Figure 11.1 in [46] and other relevant reviews [47–50]). Thus, any differences in responses might be attributed to generation examined or developmental vs. multigenerational exposure (F_1_ vs. F_2_, respectively), species (*Mus musculus* vs. *P*. *californicus*), and original dose of maternal BPA exposure. In the current study, F2 California mice pups were analyzed throughout the neonatal period, whereas in the BPA study above, F_1_ mouse pups were only assessed at PND 8 [17], which represents approximately the mid-postnatal period in mice that are generally weaned PND 21. This original study also sought to examine for other behavioral differences due to maternal vs. paternal exposure [17]. This above study tested laboratory mice where the male’s only involvement in the offspring is at the time of fertilization in terms of contribution of DNA and possible epigenetic factors [65]. These males are not involved in parental care for this species. In contrast, California mice pups rely on biparental care, as detailed in past studies [9–11]. Thus, in this latter species, it is difficult to dissect the paternal effects due to germ cells vs. that due to disrupted paternal responses. One potential way to address this issue would be to use *in vitro* fertilization and embryo transfer procedures. To date, such procedures, however, have not been performed with California mice. However, blastocysts have been derived in culture for their related cousins, *P*. *maniculatus* [66].

A previous study has shown that ultrasonic vocalizations can differ between males of different mice species [67]. It is interesting to note that the study testing effects of BPA on F_1_ vocalizations also noted increased magnitude of responses [17], which might also be attributed to reduced parental care provided by P_0_ dams directly exposed to BPA and/or developmental effects on brain regions regulating vocalization responses.

As multigenerational studies require several rounds of breeding and California mice, which produce relatively small litters, we limited ourselves to testing F_2_ pups derived from F_1_ males and females exposed to a single dose of BPA that had previously been shown to disrupt F_1_ parental care in this species [15] and leads to internal serum concentrations comparable to those detected in humans unknowingly exposed to this chemical [29, 30]. Future studies though should consider testing other doses of BPA to examine for potential non-monotonic dose responses [68].

Additional studies thus should also examine how developmental and multigenerational exposure to BPA affects molecular responses, in the brain regions, including the ventral medial hypothalamus, thalamus, central amygdala, cortex, striatum, cerebellar vermis, and nucleus ambiguous, governing pup audible and ultrasonic vocalizations [63, 69–71]. Potential hormones/genes that regulate rodent pup vocalizations and might be altered by developmental or multigenerational exposure to BPA or EE include serotonin (5-HT), forkhead box P1 (*Foxp1*), forkhead box 2 (*Foxp2*), dopamine receptor D2 (*Drd2*), neuroligin 2 (*Nlgn2*) [63, 70, 72–74]. Exposure of zebra finch male and female progeny derived from females exposed to the PCB Aroclor 1248, prior to egg-laying have significant reduction in the song control nuclei RA, which would likely compromise their song production ability in adulthood [23]. The increased song complexity in adult male starlings exposed to estrogenic EDCs is likely due to an increase in volume of the principle nucleus in the songbird brain, the high vocal center (HVC) [24].

As part of the current studies, we also examined whether maternal and paternal potentiation responses might occur after the pups are placed back with their parents and then their vocalizations responses tested again after re-isolation. In our original studies testing solely control California mice pups, we did not notice any such differences [7], although maternal potentiation responses have been reported in prairie voles, which are also monogamous and biparental [28]. When all pups are considered in the current study, the opposite effects of decreased number of syllables, suggestive of potential habituation, was observed in the second trial. However, the opposite was the case for the number of phrases that increased in Trial 2. EE pups tended to communicate more in the audible range in Trial 1 but in the ultrasonic range in Trial 2 in mid-postnatal period. Taken together, it is difficult to ascertain whether California mice pups demonstrate definitive biparental potentiation responses. Additional studies possibly testing longer periods of pup isolation might verify one way or another.

In summary, the current studies demonstrate that during specific postnatal periods, BPA and EE F_2_ pups demonstrate augmented vocalization responses. However, temporal and sex-specific differences are observed. Additionally, the responses of F_2_ EE and BPA pups were not always identical. The vocalization alterations in these groups might be due to multigenerational EDC exposure. On the other hand, increased vocalization responses by these F_2_ pups might be their attempts at signaling to their inattentive F_1_ parents to provide additional parental care. The current studies though suggest that the previously observed reduced parental care provided by F_1_ parents developmentally exposed to BPA or EE is likely not due to communication deficits by their F_2_ pups. Additional studies are needed to sort out the underlying mechanisms driving the BPA and EE F_2_ pup vocalization changes and whether it is attributed to intrinsic (e.g. neural in origin due to multigenerational exposure to these EDCs) and/or extrinsic factors (i.e. reduced F_1_ parental care).

## Supporting information

S1 Fig Median frequency calculation. A) Spectrogram showing one syllable. B) Points in the spectrogram that are above the noise level (background noise + 3 standard deviations). C) Histogram of frequencies are shown in the middle figure. The median of this distribution is the syllable’s “median frequency” and is demarcated by an "X".
(TIF)

S2 Fig Diagram of the general experimental design and split plot in space analyses. The “split plot in space” refers to the fact that the vocalization data for each pup are nested together under the dam and sire, which controls for potential litter effects. The “time” portion considers the fact that the pup vocalization responses were repeatedly measured (time of day, trial, and over the postnatal period).
(TIF)

S3 Fig Circadian effects on total number of phrases by F_2_ pups. Error bars represent SEM.
(TIF)

S1 Table Number of pups in parentheses latched onto dam prior to audio recordings in the morning and afternoon on each trial day. Total number of pups for which data were collected are listed in parentheses.
(DOCX)
