# Supplementary figures and images for: Multigenerational effects of bisphenol A or ethinyl estradiol exposure on F2 California mice (Peromyscus californicus) pup vocalizations
Source: PLoS One. 2018 Jun 18;13(6):e0199107. doi: 10.1371/journal.pone.0199107 (PMC6005501; doi:10.1371/journal.pone.0199107)

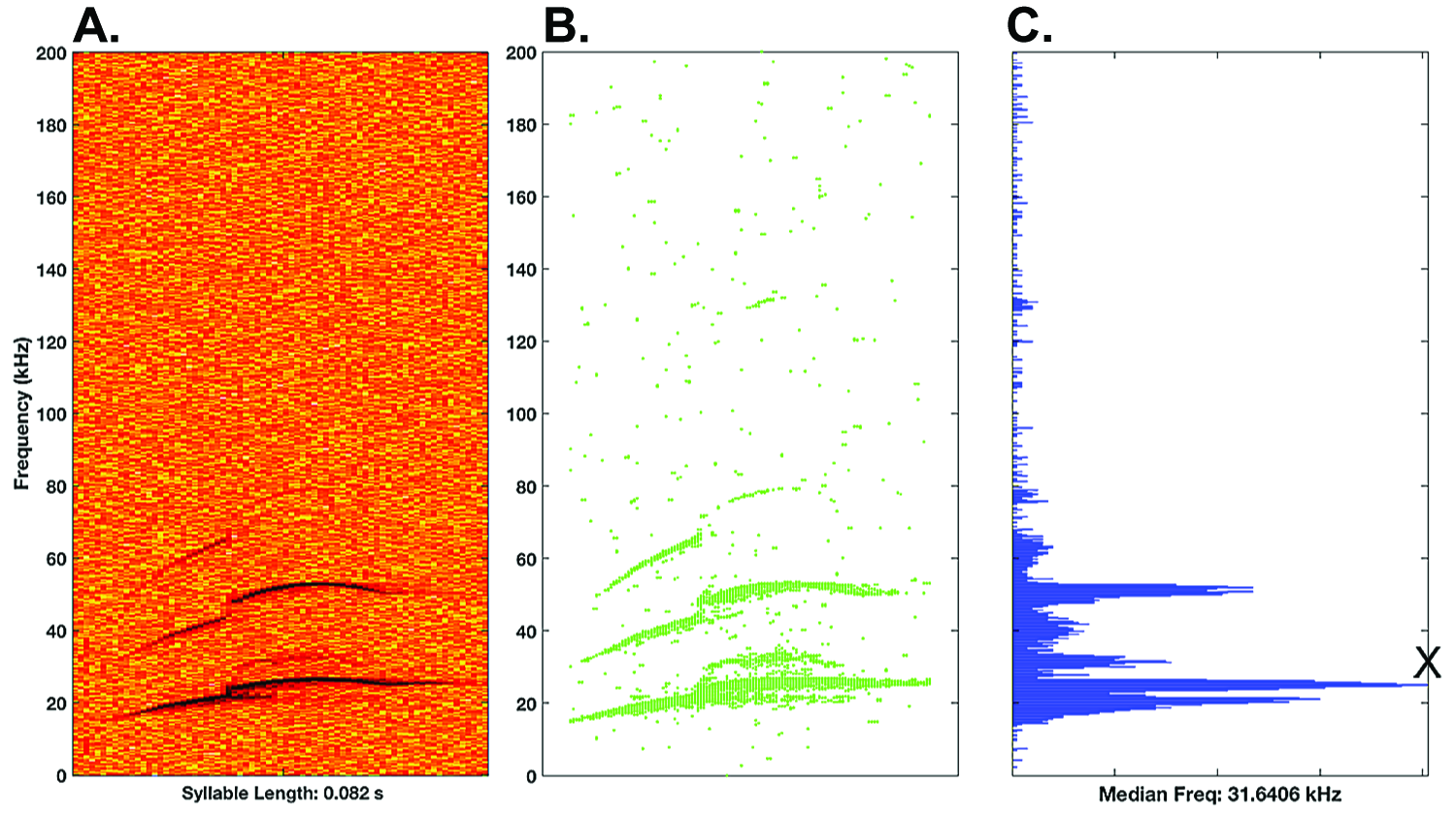

Supplement: S1 Fig — A) Spectrogram showing one syllable. B) Points in the spectrogram that are above the noise level (background noise + 3 standard deviations). C) Histogram of frequencies are shown in the middle figure. The median of this distribution is the syllable’s “median frequency” and is demarcated by an "X". (TIF) [file pone.0199107.s001.tif]

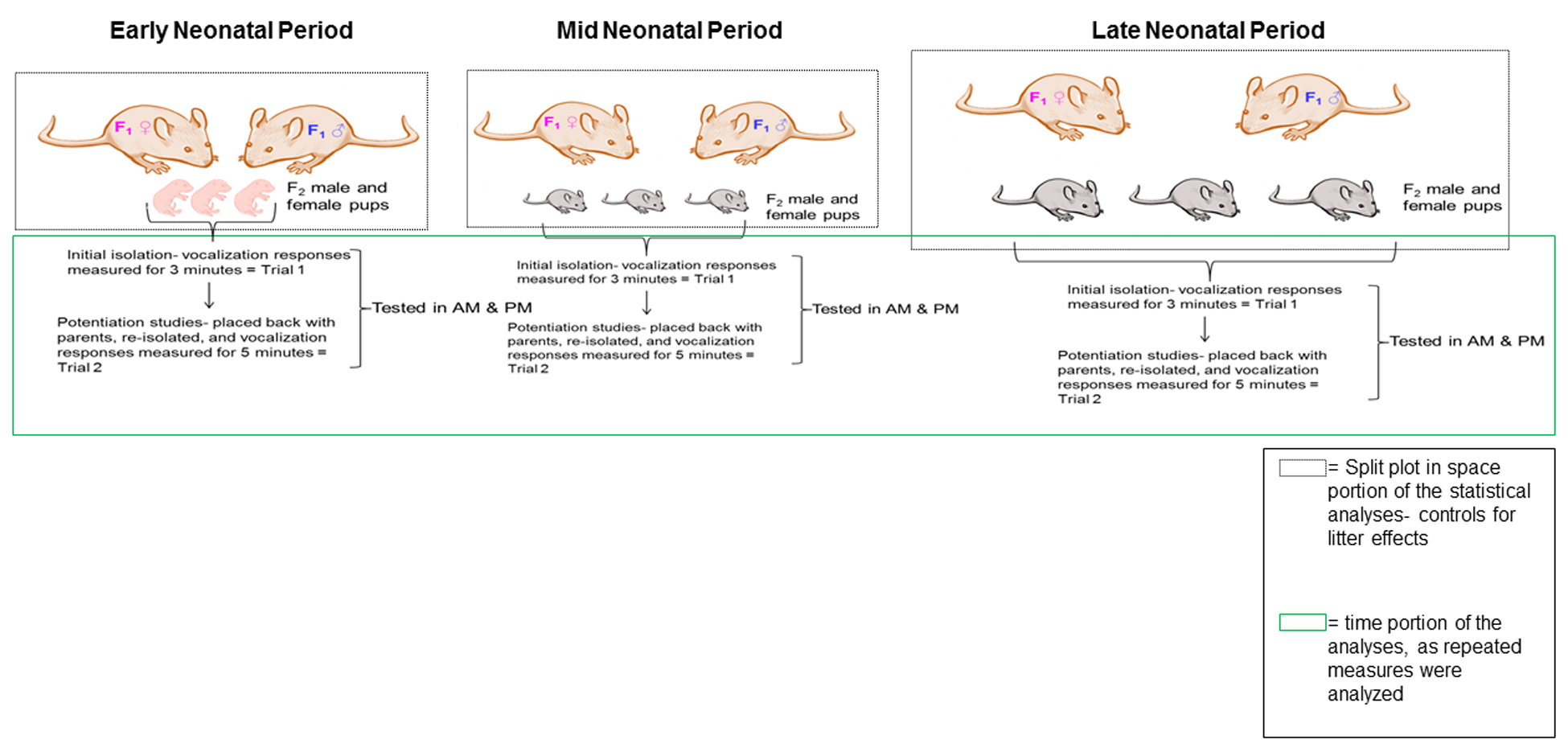

Supplement: S2 Fig — The “split plot in space” refers to the fact that the vocalization data for each pup are nested together under the dam and sire, which controls for potential litter effects. The “time” portion considers the fact that the pup vocalization responses were repeatedly measured (time of day, trial, and over the postnatal period). (TIF) [file pone.0199107.s002.tif]

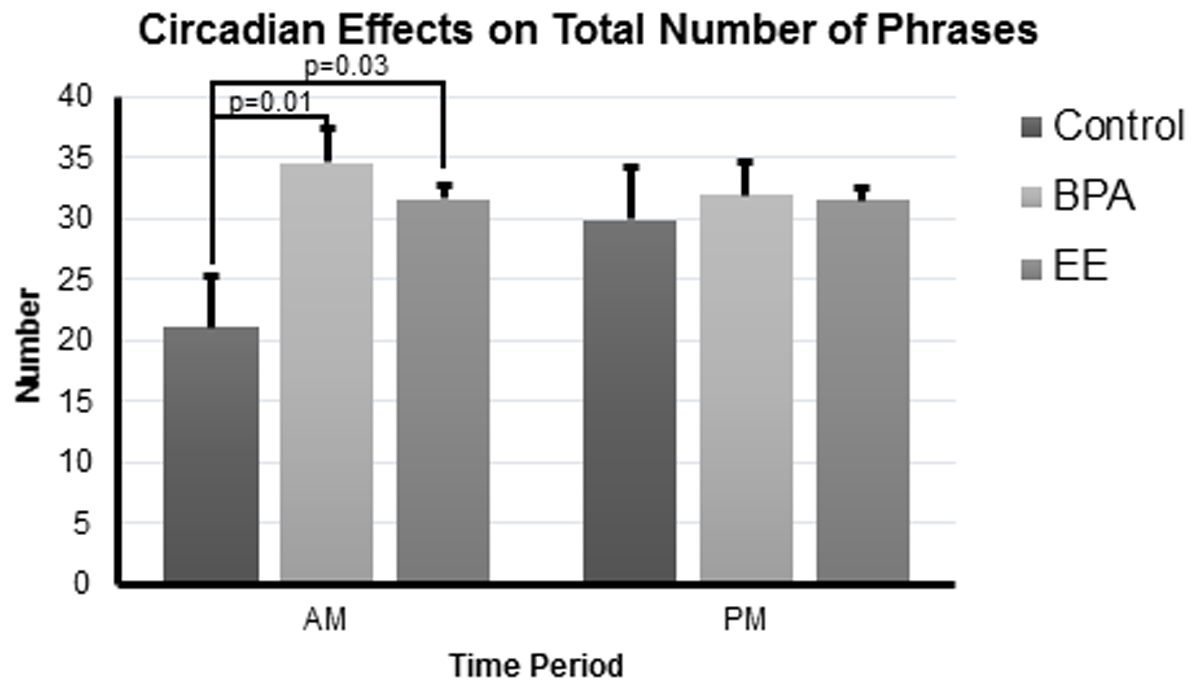

Supplement: S3 Fig — Error bars represent SEM. (TIF) [file pone.0199107.s003.tif]
