# Supplementary material for: Multigenerational effects of bisphenol A or ethinyl estradiol exposure on F2 California mice (Peromyscus californicus) pup vocalizations
Source: PLoS One. 2018 Jun 18;13(6):e0199107. doi: 10.1371/journal.pone.0199107 (PMC6005501; doi:10.1371/journal.pone.0199107)
Supplement: S1 Table — Total number of pups for which data were collected are listed in parentheses. (DOCX) [file pone.0199107.s004.docx]

| **Parental Treatment** | **PND 2-4** | | **PND 7** | | **PND 14** | | **PND 21** | | **PND 28** | |
| --- | --- | --- | --- | --- | --- | --- | --- | --- | --- | --- |
|  | AM | PM | AM | PM | AM | PM | AM | PM | AM | PM |
| **Control** | 16 (52) | 26 (52) | 3 (16) | 8 (17) | 9 (17) | 7 (17) | 8 (17) | 6 (15) | 3 (14) | 3 (14) |
| **BPA** | 61 (80) | 58 (77) | 20 (21) | 18 (22) | 21 (22) | 21 (23) | 19 (21) | 19 (23) | 12 (22) | 10 (21) |
| **EE** | 65 (96) | 68 (96) | 21 (29) | 21 (30) | 23 (31) | 22 (29) | 22 (30) | 19 (27) | 9 (27) | 15 (27) |
